# Comparison of the effects of lanthanum carbonate and calcium carbonate on the progression of cardiac valvular calcification after initiation of hemodialysis

**DOI:** 10.1186/s12872-020-01343-1

**Published:** 2020-01-30

**Authors:** Kentaro Watanabe, Hideki Fujii, Keiji Kono, Shunsuke Goto, Shinichi Nishi

**Affiliations:** Division of Nephrology and Kidney Center, Kobe University Graduate School of Medicine, 7-5-2, Kusunoki-cho, Chuo-ku, Kobe, Hyogo 650-0017 Japan

**Keywords:** Lanthanum carbonate, Calcium carbonate, Cardiac valvular calcification, Arterial plaque, Initiation of hemodialysis

## Abstract

**Background:**

Although mineral metabolism disorder influences cardiac valvular calcification (CVC), few previous studies have examined the effects of non-calcium-containing and calcium-containing phosphate binders on CVC in maintenance hemodialysis patients. The aim of the present study was to compare the effects of lanthanum carbonate (LC) with calcium carbonate (CC) on the progression of CVC in patients who initiated maintenance hemodialysis and to investigate clinical factors related to CVC.

**Methods:**

The current study included 50 subjects (mean age 65 years, 72% males) from our previous randomized controlled trial (LC group, *N* = 24; CC group, *N* = 26). CVC was evaluated as CVC score (CVCS) using echocardiography at baseline and 18 months after initiation of hemodialysis. We compared CVCS and the changes between the two groups. We also analyzed the associations between CVCS and any other clinical factors including arterial plaque score (PS) and serum phosphorus levels.

**Results:**

Baseline characteristics of study participants including CVCS were almost comparable between the two groups. At 18 months, there were no significant differences in mineral metabolic markers or CVCS between the two groups, and CVCS were significantly correlated with PS (*r* = 0.39, *p* < 0.01). Furthermore, changes in CVCS were significantly correlated with average phosphorus levels (*r* = 0.36, *p* < 0.05), which were significantly higher in high serum phosphorus and high PS group compared to low serum phosphorus and low PS group (*p* < 0.05).

**Conclusions:**

In the present study, there were no significant differences between LC and CC with regard to progression of CVC. However, serum phosphorus levels and arterial plaque seem to be important for the progression and formation of CVC in hemodialysis patients.

## Background

Cardiac valvular calcification (CVC), which includes calcification of aortic and mitral valves, as well as mitral annulus, is frequently observed in patients with chronic kidney disease (CKD); previous studies demonstrated the prevalence of CVC in maintenance hemodialysis patients to range from 23 to 68% [1–4]. In hemodialysis patients, previous studies have reported that CVC progressed rapidly and several clinical factors such as age, hemodialysis duration, uremic condition, inflammation, serum calcium levels, serum phosphorus levels, serum PTH levels, and arterial plaque were related to the CVC progression in hemodialysis patients [2, 5–10]. In hemodialysis patients, positive calcium and phosphate balance is often occurred due to mineral bone disorder and the medication such as vitamin D agents and a calcium-containing phosphate binder [11]. Vascular calcification is associated with not only passive calcium phosphate deposition but also an active cell-mediated process related to a high phosphate condition [6].

CVC is a crucial clinical problem because it is associated with an increased risk for all-cause and cardiovascular mortality in hemodialysis patients [12, 13]. Since previous studies showed that cardiovascular disease (CVD) occurred frequently during the first year after initiating hemodialysis [14, 15], clinical management, including a control of CKD-MBD, during this period is thought to be important for hemodialysis patients.

In hemodialysis patients, CKD-MBD is common and is usually treated with phosphate binders. Many studies have demonstrated that a non-calcium-containing phosphate binder had a favorable effect on vascular calcification compared to a calcium-containing phosphate binder [16, 17]. However, few previous studies examined the effects of phosphate binders on CVC in hemodialysis patients. Lanthanum carbonate (LC) is a non-calcium-containing phosphate binders with high efficacy, low pill burden, low toxicity, and is cost-effective [18].

The aim of the present study was to compare the effects of lanthanum carbonate with those of calcium carbonate (CC) on the CVC progression in patients who initiated hemodialysis and to investigate clinical factors related to CVC.

## Methods

### Study design and population

**Fig. 1:**
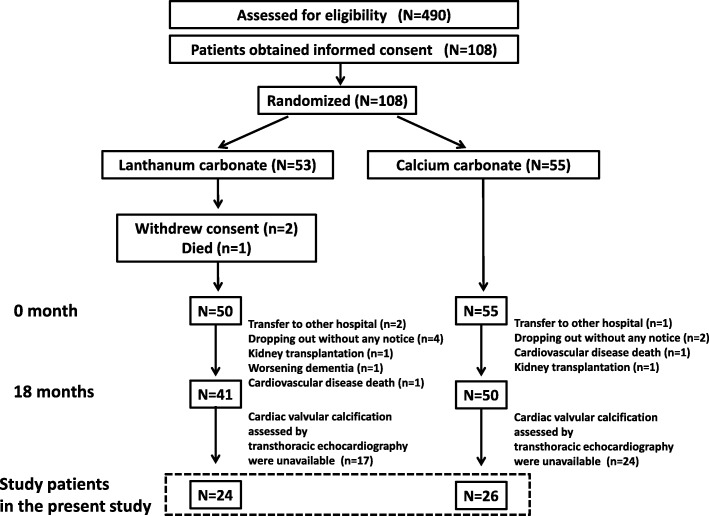
Flowchart of participants in the present study

This study was a post hoc analysis of a partial sample from our previous randomized controlled trial (RCT) in which we investigated the effects of LC on coronary artery calcification and cardiac abnormality after initiating hemodialysis [19]. Patients with contraindications to lanthanum carbonate and calcium carbonate and a history of parathyroidectomy were excluded from the previous trial. The present study included 50 patients whose CVC data were available (Fig. 1). They started inpatient hemodialysis in our hospital between December 2011 and July 2014. They were divided into the two groups: the lanthanum carbonate (LC) group (*n* = 24) and the calcium carbonate (CC) group (*n* = 26), and were treated for 18 months. Patients in each group were treated with their assigned drugs at a dose which maintained serum phosphate levels between 3.5 and 6.0 mg/dL according to the guidelines of Japanese Society of Dialysis Therapy. The administration of cinacalcet was not permitted. The administration of vitamin D agents was avoided whenever possible. When it was difficult to control serum phosphate levels within the target range by increasing a dose of CC or LC, only adding sevelamer or bixalomer was permitted. Our previous study was conducted prospectively in accordance with the Declaration of Helsinki Principles and protocols were approved by Kobe University Clinical Research Ethical Committee (approval no. 230019). Written informed consent was obtained from all individual participants included in the study. Our previous paper described the study protocol in detail [10]. In addition, to investigate the association between CVC score (CVCS) and plaque score (PS), these patients were also divided into two groups based on the severity of their PS: the low plaque score (LPS) group, PS ≤ 5 and the high plaque score (HPS) group, PS > 5. CVCS were compared between these two groups. In addition, to investigate the association between CVC score and serum phosphorus levels, we also calculated the average serum phosphate levels from baseline to 18 months (baseline, 6, 12, and 18 months) for each patient. Patients were defined as the high phosphate (HP) group or the low phosphate (LP) group depending on the median average serum phosphate levels (5.4 mg/dL).

### Echocardiographic examination

All echocardiographic examinations were performed according to the guidelines of the American Society of Echocardiography. Calcification of aortic valve, mitral valve, and mitral annulus were assessed using transthoracic echocardiography at baseline and 18 months after initiation of hemodialysis according to the evaluation method described in our previous paper [20]. We defined the presence of CVC as the presence of bright echoes of > 1 mm on any valve cusps or mitral annulus. We semi-quantitatively evaluated the severity of CVC as CVCS by counting and calculating the number of calcified valve cusps and presence of mitral annular calcification. The formula for calculation of CVCS is as follows; CVCS (0–6 points) = the number of calcified cusps in aortic valves + the number of calcified cusps in mitral valves + the presence of mitral annular calcification (no: 0, yes: 1). Left ventricular mass was calculated using the Devereux formula and was indexed for body surface area [20].

### Carotid ultrasonography examination

We evaluated the severity of carotid atherosclerosis based on the PS as evaluated previously [21, 22]. The extracranial carotid artery was divided into four segments. The first segment was the region of the internal carotid artery extending 15 mm distal to its bifurcation from common carotid artery. The second segment was the carotid bulb extending 15 mm proximal to the bifurcation. The third and fourth segment were distal and proximal common carotid artery, extending 15 to 30 mm and 30 to 45 mm proximal to the tip of the flow divider into the common carotid artery, respectively. PS was calculated by summing all the thicknesses of atheromatous plaques in the bilateral four segments of the carotid arteries in the scanning area. An atheromatous plaque was defined as a protruding lesion where intima-media thickness (IMT) was greater than or equal to 1.1 mm. The IMT was defined as the distance between the luminal-intimal interface and the medial-adventitial interface, and it was measured on a recorded longitudinal image.

### Statistical analysis

All statistical analyses were conducted using IBM SPSS statistics version 24.0 (SPSS Inc., Chicago, IL, USA). Continuous variables were expressed as mean ± standard deviation, median and interquartile range, and the differences between the groups were analyzed by Student’s t-test, paired t test, Mann–Whitney U test or Wilcoxon signed-rank test. Categorical variables were expressed as frequencies and percentage and analyzed by chi-squared test. To investigate the correlation between CVCS and clinical factors, we conducted spearman’s correlation, and multivariate ordinary regression analysis. A two-tailed *p* value of less than 0.05 was considered statistically significant.

## Results

### Patients’ characteristics

Baseline characteristics of study participants are shown in Table 1 and were almost comparable between the two groups. CKD-MBD parameters and factors related to atherosclerosis at baseline and 18 months after initiation of hemodialysis are shown in Table 2. At baseline, there were no significant differences between the two groups with regard to serum calcium levels, phosphorus levels, intact parathyroid hormone (iPTH) levels, and intact fibroblast growth factor 23 (iFGF23) levels. Low-density lipoprotein cholesterol (LDL-C) levels, C-reactive protein (CRP) level, maximum IMT, and PS were also similar between the two groups. As shown in Table 2, at 18 months, there were no significant differences in serum phosphorus levels, iPTH levels, and iFGF23 levels between the two groups. However, Serum iPTH levels were significantly decreased, and serum iFGF23 levels and PS were significantly increased in each group. Moreover, serum calcium levels increased slightly but significantly in the CC group, although not significantly different compared to the LC group. At 18 months, the number of patients treated with vitamin D agents, the other phosphate binders, and other concomitant medication were the following; vitamin D agents (LC: *n* = 20 (83%), CC: *n* = 16 (62%), *p* = 0.23), ACE-I/ARB (LC: *n* = 13 (54%), CC: *n* = 16 (62%), *p* = 0.81), statin (LC: *n* = 9 (83%), CC: *n* = 17 (65%), *p* = 0.09), warfarin (LC: *n* = 0 (0%), CC: *n* = 4 (15%), *p* = 0.14), and addition of the other phosphate binders (LC: *n* = 5 (21%), CC: *n* = 8 (31%), *p* = 0.63) (Table 3). The kind of the other additional phosphate binders were sevelamer (LC: *n* = 3 (13%), CC: *n* = 2 (8%), *p* = 0.90) and/or bixalomer (LC: *n* = 2 (8%), CC: *n* = 6 (23%), *p* = 0.30) (Table 3).

**Table 1:** Characteristics of study patients at baseline

	LC (*n* = 24)	CC (*n* = 26)	*p* value
Age (year)	66.9 ± 14.9	62.4 ± 14.4	0.27
Male (%)	21 (88)	15 (58)	0.04
BMI (kg/m^2^)	24.5 ± 3.4	23.5 ± 3.5	0.29
SBP (mmHg)	146.9 ± 18.2	148.2 ± 19.5	0.82
DBP (mmHg)	70.7 ± 13.8	75.1 ± 15.5	0.30
PP (bpm)	76.2 ± 19.1	73.0 ± 14.2	0.51
smoking (%)	16 (67)	17 (65)	1.00
Diabetes (%)	14 (58)	8 (31)	0.09
CVD (%)	4 (17)	5 (19)	1.00
Vitamin D (%)	8 (33)	8 (31)	1.00
Statin (%)	11 (46)	19 (73)	0.09
ACE-I/ARB (%)	13 (54)	16 (62)	0.81
Warfarin (%)	0 (0)	4 (15)	0.14
Hb (g/dL)	9.0 ± 1.2	8.7 ± 1.2	0.41
Alb (g/dL)	3.3 ± 0.6	3.4 ± 0.5	0.89
BUN (mg/dL)	88.3 ± 24.3	94.0 ± 27.2	0.44
Cr (mg/dL)	8.8 ± 1.9	8.5 ± 1.7	0.62
cCa (mg/dL)	8.5 ± 0.8	8.4 ± 0.8	0.73
P (mg/dL)	6.0 ± 1.3	5.9 ± 1.5	0.89
CRP (mg/dL)	0.46 ± 0.86	0.54 ± 0.74	0.75
LDL-C (mg/dL)	86.0 ± 37.2	86.8 ± 30.6	0.94
iPTH (pg/mL)	216.0 (128.5–531.8)	281.0 (217.8–422.5)	0.70
iFGF23 (pg/mL)	737.5 (456.8–1875.9)	588.1 (264.5–1880.6)	0.57
EF (%)	66.1 ± 6.0	62.1 ± 9.0	0.07
LVMI (g/m^2^)	157.5 ± 45.0	172.1 ± 53.6	0.30
IMT (mm)	1.0 (0.9–1.4)	0.9 (0.6–1.4)	0.34
PS	6.7 (3.8–14.1)	5.6 (1.3–11.6)	0.28

Values are presented as the median and interquartile or mean ± SD

*LC* lanthanum carbonate group, *CC* calcium carbonate, *SBP* systolic blood pressure, *DBP* diastolic blood pressure, *PR* pulse pressure, *CVD* cardiac vascular disease, *ACE-I/ARB* angiotensin-converting enzyme inhibitor/angiotensin II receptor blocker, *Hb* hemoglobin, *Alb* albumin, *Cr* creatinine, *BUN* blood urea nitrogen, *cCa* corrected calcium, *P* phosphorus, *CRP* C-reactive protein, *LDL-C* low density lipoprotein cholesterol, *iPTH* intact parathyroid hormone, *iFGF23* intact fibroblast growth factor 23, *EF* ejection fraction, *LVMI* left ventricular mass index, *IMT* intima-media thickness, *PS* plaque score

**Table 2:** Changes in parameters related to CKD-MBD and atherosclerosis

	LC (*n* = 24)		CC (*n* = 26)	
	0 M	18 M	0 M	18 M
cCa (mg/dL)	8.5 ± 0.8	8.8 ± 0.8	8.4 ± 0.8	9.2 ± 0.6^†^
P (mg/dL)	6.0 ± 1.3	5.5 ± 1.2	5.9 ± 1.5	5.1 ± 1.4
iPTH (pg/mL)	216.0 (128.5–531.8)	155.0 (69.1–194.5)^*^	281.0 (217.8–422.5)	98.5 (38.0–192.8)^†^
iFGF23 (pg/mL)	737.5 (456.8–1875.9)	2430.5 (750.8–7560.8)^*^	588.1 (264.5–1880.6)	2630.4 (747.1–13,741.8)^†^
LDL-C (mg/dL)	86.0 ± 37.2	84.4 ± 24.4	86.8 ± 30.6	73.6 ± 23.1^†^
PS	6.7 (3.8–14.1)	9.2 (4.8–18.0)^*^	5.6 (1.3–11.6)	7.0 (3.3–12.8)^†^

Values are presented as the median and interquartile or mean ± SD

*LC* lanthanum carbonate group, *CC* calcium carbonate, *cCa* corrected calcium, *P* phosphorus, *iPTH* intact parathyroid hormone, *iFGF23* intact fibroblast growth factor 23, *LDL-C* low density lipoprotein cholesterol, *PS* plaque score

^*^LC at 0 M versus LC at 18 M, *p* < 0.05; ^†^CC at 0 M versus CC at 18 M, *p* < 0.05

**Table 3:** Administered drugs at 18 months

	LC (*n* = 24)	CC (*n* = 26)	*p* value
Other P binders (%)	5 (21)	8 (31)	0.63
sevelamer (%)	3 (13)	2 (8)	0.90
bixalomer (%)	2 (8)	6 (23)	0.30
Vitamin D (%)	20 (83)	16 (62)	0.23
Statin (%)	9 (38)	17 (65)	0.09
ACE-I/ARB (%)	13 (54)	16 (62)	0.81
Warfarin (%)	0 (0)	4 (15)	0.14

*LC* lanthanum carbonate group, *CC* calcium carbonate, *Other P binders* Other phosphate binders, *ACE-I/ARB* angiotensin-converting enzyme inhibitor/angiotensin II receptor blocker

### Effects of LC and CC on CVC progression

**Fig. 2:**
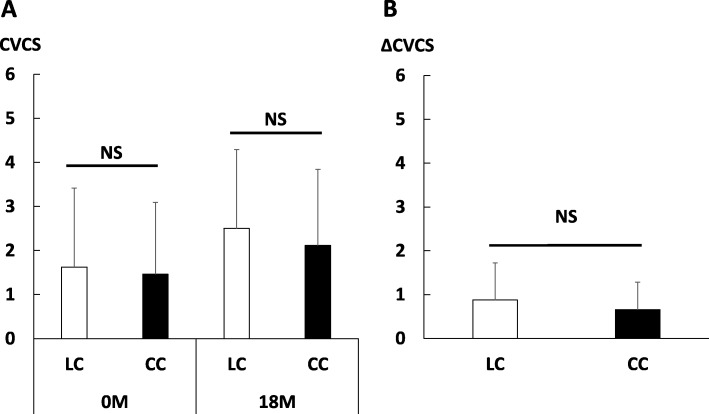
CVCS in the LC or CC group. **a** Total CVCS at baseline and 18 months. **b** Change in total CVCS from baseline to 18 months. Bars indicate the means and error bars indicate the standard deviation (SD). CVCS, cardiac valvular calcification score; LC, lanthanum carbonate group; CC, calcium carbonate; NS, not significant

As shown in Fig. 2a, there were no significant differences in CVCS at baseline between the two groups. Although CVCS increased significantly at 18 months in each group (LC: *p* < 0.001; CC: *p* < 0.001), there were no significant differences in CVCS at 18 months, nor were there changes in CVCS from baseline to 18 months (Fig. 2a, b).

### Correlation of CVCS with clinical factors

CVCS at baseline tended to correlate with PS at baseline (*r* = 0.27, *p* = 0.06), and CVCS at 18 months was significantly correlated with PS at 18 months (*r* = 0.39, *p* < 0.01). In addition, the changes in CVCS from baseline to 18 months were not correlated with changes in PS from baseline to 18 months; however, the changes in CVCS from baseline to 18 months were significantly correlated with the average serum phosphorus levels (*r* = 0.36, *p* < 0.05) or age (*r* = 0.28, *p* < 0.05).

To elucidate clinical factors related to change in CVCS, we performed multivariate regression analysis including the average serum calcium levels, the average serum phosphorus levels, PS at baseline, and age. As the sample size of the present study was relatively small, we could not choose so many variables in multivariate analysis. Therefore, age, serum calcium levels, serum phosphate levels, and PS, which played an important role in the CVC progression and significantly associated with CVC, were included in the analysis. Age is a common risk factor for calcification and serum calcium and phosphate levels are also important risk factors particularly in hemodialysis patients. In fact, the results of our study showed that age, serum phosphate levels, and PS were significantly associated with CVCS. The results of multivariate analysis showed that the average serum phosphorus levels (odds ratio: 3.71, 95% confidential interval: 1.71–8.05) and age (odds ratio: 1.09, 95% confidential interval: 1.02–1.15) were significant and independent predictors for the changes in CVCS from baseline to 18 months.

### Correlation of CVC progression with serum phosphorus levels and PS

**Fig. 3:**
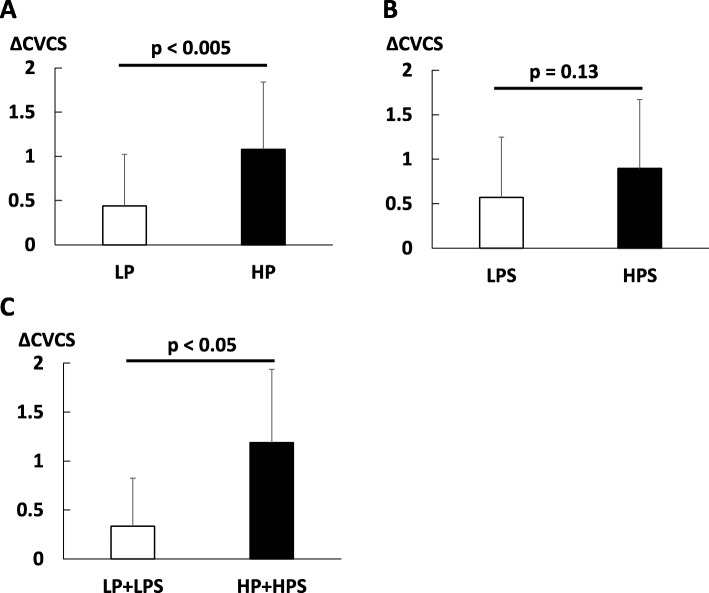
CVCS based on average serum phosphate levels and the amount of plaque. **a** Changes in total CVCS from baseline to 18 months in the LP and HP group. **b** Changes in total CVCS from baseline to 18 months in the LPS group and in the HPS group. **c** Changes in total CVCS from baseline to 18 months in the LP + LPS and HP + HPS group. Bars indicate the means and error bars indicate the standard deviation (SD). CVCS, cardiac valvular calcification score; LP, low phosphorus group; HP, high phosphorus group; LPS, low plaque score group; HPS, high plaque score group

The changes in CVCS from baseline to 18 months were significantly greater in the HP group than in the LP group (Fig. 3a). Similarly, changes in CVCS from baseline to 18 months tended to be greater in the HPS group than in the LPS group (Fig. 3b). The changes in CVCS from baseline to 18 months were significantly greater in the HP + HPS group than in the LP + LPS group (Fig. 3c).

## Discussion

The present study demonstrated that (1) there were no significant differences in CVCS at 18 months and the changes in CVCS from baseline to 18 months between the LC and CC groups; (2) CVCS were almost correlated with PS at baseline and were significantly correlated with PS at 18 months; (3) serum phosphorus levels and age were significant predictors of changes in CVCS; (4) changes in CVCS tended to be greater in the HPS group than in the LPS group and were significantly greater in the HP group than in the LP group; (5) the change in CVCS from baseline to 18 months was significantly greater in the HP + HPS group than in the LP + LPS group.

Many studies have shown that serum phosphorus has a great impact on vascular calcification and is related to CVC [23–28]. Therefore, the control of serum phosphorus levels by phosphate binders is an important strategy for attenuating the formation of CVC. In the present study, serum phosphorus levels did not differ between the LC and CC groups and was kept within a target range. Therefore, there might be no differences in CVC progression between the LC and CC groups. However, the present study also showed that serum phosphorus levels were very important for CVC progression because the changes in CVCS were significantly associated with serum phosphorus levels regardless of the types of phosphate binders. In addition to clinical study, in vitro studies revealed that extracellular phosphate is transported via sodium-dependent phosphate transporters and upregulates osteogenic genes, thereby being consider to cause vascular calcification [6, 29].

Calcium-containing phosphate binders can induce oral calcium loading and a previous study showed oral calcium loading was associated with CVC [30], suggesting that a calcium-containing phosphate binder can affect CVC. Raggi et al. conducted a RCT to determine differences in the effects of sevelamer hydrochloride and calcium acetate on CVC in maintenance hemodialysis patients [31]. Their study showed that 52 weeks after treatment, the progression of aortic valvular calcification did not differ between the two groups, but mitral valvular calcification progressed more in those treated with calcium acetate compared to those treated with sevelamer hydrochloride, although not statistically significant. However, the actual serum calcium levels were not described in their manuscript; in the current study, serum calcium levels did not significantly differ between groups despite oral calcium loading in the CC group and we did not find a significant association with serum calcium levels and CVC.

Medication regimens of CKD-MBD other than phosphate binders include calcimimetics and active vitamin D agents which can also affect CKD-MBD parameters. Previous work has shown that treatment with cinacalcet and low-dose vitamin D agents, which are medication to prevent secondary hyperparathyroidism, reduced CVC progression compared to treatment with only vitamin D agents [32]. However, since serum calcium, phosphorus, and PTH levels were also lower in those treated with cinacalcet and low-dose vitamin D agents compared to only vitamin D, these changes in the CKD-MBD parameters could subsequently affect the progression of CVC. In contrast, in the present study, serum PTH, calcium, and phosphorus levels were similar between the two groups and within a target range in both groups.

Taken together, we speculate from these data that one of the reasons we did not observe a significant difference in CVCS between the LC and CC groups was because of similar and well-controlled CKD-MBD parameters, including the serum calcium and phosphorus levels. In addition, it was suggested that the number of study subjects was too small to detect statistically differences, which may have contributed to no difference in CVCS between the LC and CC groups.

Interestingly, the present study showed that the HPS group tended to have greater CVCS and changes in CVCS than the LPS group, and also that PS was correlated with CVCS, although there was no significant correlation between changes in CVCS and changes in PS. These results showed that it is not the changes in plaque but the amount of plaque which was associated with CVC. In other words, plaque was associated with the formation of CVCS rather than the progression of CVCS. Previous studies also revealed that arterial plaque was related to existence of CVC in patients with CKD [33], and histopathological studies of aortic valvular sclerosis showed focal subendothelial plaque-like lesions on the aortic side of the leaflet [8]. Our previous study revealed that the plaque composition of coronary culprit lesions changed from necrotic core-rich to extensively calcium-rich plaques with declining kidney function [34].Considering these results, if arterial plaque indeed reflects plaque lesions of cardiac valves, it is suggested that the existence of plaque lesions interact with CKD-MBD and are thus closely associated with CVC formation. Dyslipidemia is also related to the plaque formation and is a crucial risk factor for CVC progression [28]. In previous studies, treatment with statin reduced the progression of CAC, carotid IMT, and the volume of coronary atheroma [35]. However, it is reported that treatment with statin did not suppress CVC progression [36] and the results of our study was not able to demonstrate a significant association between CVCS and lipid profile. From these data, we speculate that we may have to treat with statin before calcification occur.

These results suggest that several factors are related to CVC formation and progression in hemodialysis patients. In particular, hyperphosphatemia may be related to the progression of CVC and the amount of plaque may be related to the formation of CVC. To reduce the formation and progression of CVC, we should intervene several factors related to CVC simultaneously. Therefore, we suggest that a combination therapy with statin and phosphate binders might halt CVC progression.

There are several limitations in this study. First, there might be the existence of type two error. It is possible that sample size is relatively small to detect the significant differences in CVC progression between LC and CC. Second, the present study has bias due to the study design including only partial subjects in our previous RCT. These might affect the result of the present study.

## Conclusions

In the present study, the effects on the progression of CVC of LC did not significantly differ from that of CC. However, serum phosphorus levels were significantly associated with CVC progression. Similarly, arterial plaque formation was also associated with the formation of CVC. We speculate that control of hyperphosphatemia and plaque formation may be important factors in reducing CVC formation and progression. Further studies are needed to elucidate the detailed mechanisms of formation and progression of CVC.

## Data Availability

The datasets generated and/or analyzed during the current study are not publicly available due to patient confidentiality but are available from the corresponding author on reasonable request.

## Abbreviations

ACE-I/ARB: Angiotensin-converting enzyme inhibitor/angiotensin II receptor blocker
CC: Calcium carbonate
CKD: Chronic kidney disease
CKD-MBD: Chronic kidney disease-mineral bone disorder
CRP: C-reactive protein
CVC: Cardiac valvular calcification
CVCS: CVC score
CVD: Cardiovascular disease
HP: High phosphate
HPS: High plaque score
iFGF23: Intact fibroblast growth factor 23
IMT: Intima-media thickness
iPTH: Intact parathyroid hormone
LC: Lanthanum carbonate
LDL-C: Low-density lipoprotein cholesterol
LP: Low phosphate
LPS: Low plaque score
PS: Plaque score
RCT: Randomized controlled trial
